# Sharing With Patients the Uncertainties Regarding the Management of Dyspepsia

**DOI:** 10.3389/fmed.2021.681587

**Published:** 2021-09-16

**Authors:** Jochanan Benbassat

**Affiliations:** Department of Medicine, Hebrew University - Hadassah Medical Center, Jerusalem, Israel

**Keywords:** dyspepsia, peptic ulcer, gastric cancer, lead time, endoscopy, mass screening

## Abstract

**Background:** The management of patients with dyspepsia is uncertain. Some authors advocate endoscopy for all; others restrict endoscopy only to patients at high risk of gastric cancer, namely to those above an age threshold, or with a family history, dysphagia, loss of weight, anemia, or a childhood in Asian countries. Still others recommend various combinations between test-and-treat for Helicobacter pylori, anti-secretory treatment, and/or endoscopy.

**Objective:** To highlight the uncertainties in the choice between the various strategies and argue that these uncertainties should be shared with the patient.

**Method:** An overview of reported life expectancy, patient satisfaction, gastric cancer detection rates, symptom relief, and cost effectiveness of the management strategies for dyspepsia.

**Main Findings:** There are no randomized controlled trials of the effect of screening by endoscopy on mortality of patients with gastric cancer. Lower grades of evidence suggest that early diagnosis reduces this mortality. Analyses, which assume a survival benefit of early diagnosis, indicate that mass screening in countries of high incidence gastric cancer (> 10 cases per 100,000) and targeted screening of high-risk persons in countries of low-intermediate incidence (<10 cases per 100,000) is cost-effective at a willingness to pay of $20,000–50,000 per QALY. Prompt endoscopy appears to be best for patient satisfaction and gastric cancer detection, and test-and-treat for H pylori—for symptom relief and avoiding endoscopies.

**Conclusions:** The gain in *life expectancy* is the main source of uncertainty in the choice between management strategies. This choice should be shared with the patients after explaining uncertainties and eliciting their preferences.

## Introduction

An estimated 20% of the population experience epigastric discomfort without reflux referred to as dyspepsia (1). A 2010 review of the literature indicated that, in the West, dyspepsia is associated with “non-ulcer” or “functional” dyspepsia (73%), erosive esophagitis (13.4%), peptic ulcer (8%) and gastric cancer (0.4%) (2). Gastric cancer and peptic ulcer are associated with Helicobacter pylori (H pylori) infection, and its eradication has been claimed to reduce the relative risk of gastric cancer (RR = 0.67) (3), and to have prevented 18,665 peptic ulcer deaths in Australia between 1990 and 2015 (4). Yet, although about half of the world's population is infected, only a small proportion of people develop cancer and peptic ulcer; although both related to H. pylori infection, gastric cancer, and duodenal ulcer seem to be mutually exclusive (5); and during the last decades, there has been an unexplained worldwide decline in their prevalence (6–8) with the decline in peptic ulcer and gastric cancer preceding that of H pylori (9).

The prevalence of disorders associated with dyspepsia is subject to geographic and socio-economic variations. In Asia, gastric cancer occurs in as many as 1.3% of all dyspeptic patients (10); in the UK, peptic ulcer was found in 4.7% of the highest social class and 17.1% in the lowest class (11), and the prevalence of H pylori is higher in less affluent regions of Europe (6). Dysphagia (OR 3.1), weight loss (OR 2.6), and age >55 years (OR 9.5) predict cancer in dyspeptic patients (12). A serum pepsinogen I / pepsinogen II ratio of <3.0 has 85% sensitivity and 74% specificity for cancer (13), and a serum level of trefoil factor 3 (a protein secreted by the gastrointestinal tract) of 3.6 ng/mL or more had 81% sensitivity and 81% specificity for cancer (14). The various guidelines for management of dyspepsia have used these tests and risk indicators in order to identify patients at high risk for gastric cancer.

For patients with dyspepsia as the primary symptom, the American College of Gastroenterology (ACG) and Canadian Association of Gastroenterology (CAG) recommend prompt endoscopy for those older than 60 years (conditional recommendation; very low-quality evidence). For younger patients the guidelines recommend H pylori test-and-treat and proton pump inhibitor therapy for those who test negative to H pylori or do not respond to its eradication (strong recommendations; high-quality evidence) (1). Others advocate endoscopy for all, including those at low risk of malignancy (15). Still others recommend testing for H. pylori and pepsinogen levels; eradication therapy and follow up by annual and bi-annual endoscopy for those who test positive for either H. pylori infection or pepsinogen levels or both (16). The objective of the following analysis is first, to identify the uncertainties in the choice between these strategies and second, to argue that these uncertainties should be shared with the patient and eliciting his/her preferences.

## Uncertainties in the Choice Between Management Strategies for Dyspepsia

### Life Expectancy

The main uncertainty in the choice of management of dyspeptic patients is the inconsistency in their reported life expectancy. On the one hand, a 2010 systematic review of the literature indicated that the prevalence of gastric cancer among patients with dyspepsia was similar to that of subjects without dyspepsia (2), and dyspepsia was not associated with higher mortality during a 10 year follow up (17). On the other hand, a 2016 review did identify studies that found a higher prevalence of gastric cancer in dyspeptic patients with heartburn, regurgitation, epigastric pain, postprandial fullness, early satiety, abdominal bloating, nausea, dysphagia, vomiting (18), and there is evidence that eradication of H. pylori and endoscopic screening may reduce mortality from peptic ulcer (4) and gastric cancer (19).

There are no randomized controlled trials of the effect of detection of peptic ulcer in asymptomatic persons on mortality. Most published analyses assume that the prognosis of an endoscopic finding of peptic ulcer in dyspepsia is the same as that in patients with clinically overt peptic ulcer. However, such finding may have no clinical significance (18), and therefore the efficacy of its early treatment in preventing complications and mortality of peptic disease remains uncertain. The undisputed benefit of H pylori eradication precludes performance of prospective long-term comparisons of treated and untreated asymptomatic patients with endoscopically detected peptic ulcer.

Similarly, there are no randomized controlled trials of the effect of early detection of gastric cancer on mortality. In Korea, comparison between patients with gastric cancer who participated in the national cancer screening program at least once with those who did not participate indicated that the program increased 5-year *survival* from 62 to 78% (20). A 2018 review of cohort studies and case-control studies indicated that screening of Asian individuals reduced by 40% the risk of gastric cancer *mortality* (19). However, these findings may have been affected by biases, such as lead time bias, i.e., the interval from presentation with dyspepsia to development of symptoms of gastric cancer.

### Patient Satisfaction, Cancer Detection, and Symptom Relief

A 2019 systematic review of randomized controlled trials assessed the effectiveness of five management strategies in dyspeptic patients aged ≥18 years: prompt endoscopy, “test-and-treat” for H pylori, “test and scope” (test for H pylori; endoscopy for those testing positive; acid suppression for those testing negative), empirical acid suppression, and symptom management. The outcomes of interest were developing symptoms at follow-up, likelihood of receiving endoscopy, satisfaction with management and gastrointestinal cancer detection. It was found that the best strategy was prompt endoscopy, if the outcome measures were patient satisfaction or gastrointestinal cancer detection rates, while the best strategy was test-and-treating for H pylori, if the outcome measures were symptom relief and number of endoscopies avoided (21).

### Cost Effectiveness

Decision analyses have been used to explore the costs and benefits of endoscopic mass screening and of H pylori test-and-treat. A 2020 systematic review of such analyses revealed that, in countries of high incidence gastric cancer (> 10 cases per 100,000) and targeted screening of high-risk groups within otherwise low-intermediate incidence (10 cases or less per 100,000) countries were cost-effective at a willingness to pay of about $20,000–50,000 per QALY. Endoscopic screening of individuals younger than 50 years in high incidence countries and of populations in low-intermediate incidence countries was not. Most decision models assumed that, compared with no screening, endoscopic screening reduced gastric cancer mortality (22).

A 2020 cost-effectiveness analysis compared three strategies: “Test and treat” for H pylori, gastrointestinal endoscopy and symptomatic treatment for dyspepsia relief within 4 weeks and “peptic ulcer avoided” and “gastric cancer avoided” within 10 years. The analysis indicated that for dyspepsia relief, “test and treat” was the most cost-effective strategy (883€/success) compared with endoscopy (1628€/ success) and symptomatic treatment (990€/success). For the endpoint “peptic ulcer avoided,” test and treat was the most cost-effective strategy (421€/peptic ulcer avoided/y) compared with endoscopy (728€/peptic ulcer avoided/y) and symptomatic treatment (632€/peptic ulcer avoided/y). For the endpoint “gastric cancer avoided,” test and treat was the most cost-effective strategy (524€/gastric cancer avoided/y) compared with endoscopy (716€/gastric cancer avoided/y) and symptomatic treatment (696€/gastric cancer avoided/y) (23). Another decision analytic model indicated that, when compared with no screening, an H pylori screen-and-treat strategy in asymptomatic Chinese at the age of 20 years saved 288.1 million dollars, 28,989 life years, and 111 663 QALYs, and prevented 11 611 gastric cancers, 5,422 deaths from gastric cancer, and 1,854 deaths from peptic ulcer during life (24).

## Discussion

Two main findings emerge from the presented non-systematic overview. First dyspepsia is a common clinical problem; second, its management is wrought with uncertainties. Since the 1970s, the recognition of patients' right to participate in decisions about their care has promoted a shared decision making (SDM) consultation style, whereby patients convey their concerns, preferences and knowledge about their problem, while doctors provide explanations about available courses of action (25).

I believe that the point of departure of SDM should not be acknowledging that a decision is required and communicating uncertainty (26), but rather identifying the patient' concerns. In-depth interviews have indicated that patients expect their doctor to apply his/her knowledge to the specifics of their individual cases, and were disappointed when doctors cited only prognostic statistics (27). To meet this expectation, doctors should balance between standardized approaches to management and individual patient concerns and preferences. Identifying patients' concerns requires recognition that they may not be directly expressed (28), and that their elucidation may require questions, such as “*of all you told me, what makes you worry most?*,” *or “What do you want most to avoid?”* It is also important to discern between patients who prefer a passive relationship with their doctor, and those who are hesitant to ask question, although they prefer to be involved in their treatment. To achieve this distinction, a doctor may ask “*Before I answer your questions, it would help me if you told me what you already know about your disease.”* Patients may respond by expressing their concerns (“*I hope that it is a transient indigestion; however, I dread the possibility of cancer”*). If the patient does not respond to the doctor's prompt (“*I don't have the slightest idea”*), the doctor may ask: “*I am very interested to have your opinion how we should proceed*,” or “*Do you want me to tell you my thoughts about your disease and the various options of its further investigation/treatment*.” The patient's answer (“*please just tell me what to do”* or “*yes, tell me what these options are*”) will probably make explicit his/her preferences about involvement in SDM.

The main area of uncertainty in the choice of a management strategy in dyspeptic patients is the benefit incurred by early treatment of gastric cancer. So far, this benefit has been derived from cohort and case-control studies of mortality (19), from comparisons between the survival of screened and not-screened patients with gastric cancer (20), and from cost-effectiveness analyses of the efficacy of test-and-treat for H pylori in preventing peptic ulcer and gastric cancer (23, 24). However, these studies may be confounded first, by possibly erroneous assumptions that the natural history of peptic ulcers and gastric cancers detected by endoscopy in dyspeptic or asymptomatic patients is the same as in patients with clinically overt disease, and second, by lead time bias. Hence the need of randomized controlled trials in order to confirm the reduced mortality after screening for gastric cancer. Pending such trials, dyspeptic patients should be offered test-and-treat for H pylori, while the decision for endoscopy should be guided by their preferences. After receiving an explanation of available data, some patients may consider the gain incurred by endoscopy too small to justify its inconvenience, while others may prefer endoscopy in order to relieve their fear of malignancy.

## Author Contributions

The author confirms being the sole contributor of this work and has approved it for publication.

## Conflict of Interest

The author declares that the research was conducted in the absence of any commercial or financial relationships that could be construed as a potential conflict of interest.

## Publisher's Note

All claims expressed in this article are solely those of the authors and do not necessarily represent those of their affiliated organizations, or those of the publisher, the editors and the reviewers. Any product that may be evaluated in this article, or claim that may be made by its manufacturer, is not guaranteed or endorsed by the publisher.
